# A pilot self-coach alignment audit in NCAA collegiate rowers: field feasibility, null findings, and design-sensitivity analysis for future self-verification research

**DOI:** 10.3389/fspor.2026.1902953

**Published:** 2026-08-25

**Authors:** Zacharias Papadakis, Andreas Stamatis

**Affiliations:** 1 Department of Health Sciences & Clinical Practice, Barry University, Miami Shores, FL, United States; 2 Department of Health & Sport Sciences, University of Louisville, Louisville KY, United States; 3 Sports Medicine Institute, University of Louisville Health, Louisville, KY, United States

**Keywords:** measurement stability, null results, small sample size, statistical power, underpowered studies, mental toughness

## Abstract

**Background:**

Testing relational mechanisms like self-verification (SV) in competitive collegiate sport is constrained by field-feasible measurement and the small-N problem. We introduce a brief alignment audit and evaluate its field feasibility and statistical behavior in a single-team pilot

**Methods:**

NCAA Division II women rowers (*N* = 12) completed four in-season waves aligned with 2,000-m ergometer assessments, reporting self-rated mental toughness (MT) via the Mental Toughness Index (MTI), meta-perceptions of head and assistant coach appraisals, and their preferred coach for feedback (self-reported, not observed). Because coach preference was perfectly invariant within every athlete across all four waves, the design was analytically reduced to a cross-sectional athlete-level comparison (*N* = 12); GEE and GLMM models were retained only as sensitivity diagnostics, since quasi-complete separation precluded their use for primary inference. The perceived-proximity proxy, *Δ*Alignment, was the difference between absolute self–coach discrepancies, a coarse proxy that cannot distinguish self-verification from self-enhancement or structural coach-role preference.

**Results:**

Head-coach preference was stable at 33.3% across all 48 observations. Athlete-level analyses converged on a null: logistic regression returned OR = 1.01 [95% CI (0.71, 1.56), *p* = .950]; Welch *t*(3.68) = 0.04, *p* = .968; Hedges’ *g* = 0.03 [95% CI (−1.08, 1.14)]; Fisher exact *p* = .236 for alignment directionality. The 95% CI width of 2.22 Hedges’ g units is the primary index of design insensitivity. Temporal stability of *Δ*Alignment was moderate to good [ICC(2,1) = .73, 95% CI [.48,.90]]. GEE and GLMM point estimates were uniformly null (all ORs = 1.00). Tiered simulation-based power analysis at *N* = 12 was flat across OR = 1.5–3.0 (13.5–13.9%), confirming the design's insensitivity to effect size rather than absence of effect.

**Conclusions:**

Perceived self–coach alignment was not detectably associated with self-reported coach preference. This reflects the inability of a single-team, *N* = 12 design to provide precise information, not evidence against SV theory. The primary contribution is methodological: a reproducible, low-burden audit template with open-science materials (data, code, RSA-ready component scores at OSF) and a design-sensitivity framework to guide prospectively powered multi-team replication.

## 1 Introduction

The research–practice gap in sport psychology has pushed the field toward tools that are ecologically valid and statistically defensible. Calls to close this divide have grown louder (1), with frameworks urging co-production and theories that are practically concise and faithful to what coaches actually do (2, 3), a gap frequently termed the “valley of death” (4). Professional bodies now promote funding models that pair researchers with practitioners (5–7), underscoring the necessity for scientific tools that fit inside the time constraints of competitive sport without sacrificing scholarly rigor (8–10).

Competitive collegiate sport science amplifies this challenge. The very nature of high-performance environments, limited rosters, regulated schedules, and protected access, creates a fundamental tension between capturing authentic competitive dynamics and achieving the statistical power needed for robust inference (11–14). Deploying psychological diagnostics in competitive settings such as NCAA women's rowing, a population underrepresented in psychological research (15, 16), offers high ecological validity but exposes two fundamental challenges. The first is the small-N constraint: fixed rosters cap statistical power and replicability (11, 13, 17, 18). The second is the analytical challenge posed by congruence-based relational hypotheses: small samples, limited outcome variation, and crude discrepancy scores can make it difficult to determine whether perceived alignment is meaningfully associated with coach preference (19–22).

*Self-verification* (SV) theory posits that people seek interaction partners who confirm their stable self-views; critically, it is the athlete's perceived appraisal of the coach, not the coach's actual appraisal, that drives this preference (23–25). Activating SV processes requires that the evaluative domain be sufficiently central to the individual's self-concept. *Mental toughness* (MT) is a plausible candidate domain for competitive athletes: Gucciardi (26) frames MT explicitly as an achievement-oriented self-belief system embedded within athletes’ characteristic adaptations and narrative-identity layers, and Coulter et al. (27) situate MT across trait, characteristic adaptation, and narrative identity layers using McAdams’ three-domain personality model (28) — potential corresponding to the self-concept levels at which SV strivings are theorized to operate (24).

Mental toughness is additionally well suited to this design because it can be assessed briefly and repeatedly in-season via the concise, validated Mental Toughness Index (MTI) (26, 29, 30), and because SV processes are thought to activate most strongly when self-views are held with confidence. In this study, we operationalized MT's centrality to self-concept as a theoretical assumption rather than an established fact, since classical SV work was developed around global self-esteem rather than a domain-specific construct. This distinction is theoretically important because SV and self-enhancement may plausibly coexist in competitive sport: athletes may prefer a coach whose appraisal seems accurate, but they may also prefer one whose appraisal is simply more favorable, and the present design cannot distinguish between these motivational processes (24).

To address these constraints, this study introduces a brief “alignment audit” designed for in-season use. Mental toughness functions here as the evaluative content through which a self-coach alignment audit can be developed, rather than as the primary outcome; what matters for SV is not what the coach actually thinks but what the athlete believes the coach thinks (25, 31–35). The study implements this audit across four in-season competitive waves, using analytical methods suited for small, clustered samples alongside a simulation-based design-sensitivity analysis to frame what the data can and cannot support (36–40). It also addresses three specific gaps in the literature: the scarcity of field-based SV evidence in sport, the underrepresentation of female collegiate rowers in relational psychology research, and the limited reach of relational, longitudinal designs in MT work, which has leaned heavily on cross-sectional and male samples (34, 41–44). As a whole, the study responds to calls for translational tools that carry theoretical rigor into applied coaching practice (45, 46).

This pilot study investigated the feasibility of a brief self-coach alignment audit and its cross-sectional association with self-reported coach preference in a single NCAA roster. Specifically, we examined whether *Δ*Alignment, the difference between absolute self–coach discrepancies, showed any detectable association with self-reported coach preference after accounting for 2 K performance. Because *Δ*Alignment cannot separate SV from self-enhancement or structural coach-role preference, it was treated as a coarse perceived-proximity proxy rather than a direct measure of SV (24, 47). Based on SV theory, we expected that greater perceived proximity to a coach would be directionally associated with preference for that coach. Given the single-team sample size, this expectation was treated as a design-feasibility question rather than a theory-confirming hypothesis.

## 2 Methods

### 2.1 Study design

This study used a repeated-measures observational design spanning one NCAA competitive season, intended to capture within-person preference dynamics. Coach preference proved invariant within all 12 athletes across all four waves; the analytic consequences of this finding are addressed in detail in §3. Statistical Analysis. Data were collected at four waves, each scheduled approximately three weeks apart to coincide with existing NCAA-mandated race and team's scheduled testing dates capturing psychological dynamics across preseason conditioning, mid-season racing, and championship preparation while minimizing disruption to training (16, 48–55).

At each wave, athletes completed the protocol in sequence. They first performed a maximal 2,000-meter (2 K) ergometer time trial (15, 56, 57). Under standardized administration conditions immediately after, they completed three MTI administrations; self-appraisal, perceived head coach appraisal, and perceived assistant coach appraisal followed by a discrete coaching preference choice (58–65).

### 2.2 Participants

Participants were the entire varsity roster (*N* = 12) of an NCAA Division II women's rowing program (age: 20.75 ± 2.14 years; rowing experience: 2.83 ± 3.07 years). These data were collected as part of a broader within-season monitoring protocol involving this roster; a related analysis examining within-athlete associations between mental toughness and 2 K ergometer performance in this same cohort has been reported elsewhere (66). This census-based sampling approach limits broad generalizability but maximizes roster-level coverage and ecological validity and is a recognized approach for specialized, roster-restricted athletic populations (11, 36, 67, 68). All athletes were healthy and cleared for full competitive participation. The university's Institutional Review Board approved the study (IRB #1968553-3, 11/06/2022), which was conducted in accordance with the Declaration of Helsinki. Each athlete provided written informed consent after being informed of the study's objectives, procedures, and right to withdraw.

### 2.3 Procedures

#### 2.3.1 2 K rowing performance

Each athlete performed a maximal 2 K time trial on a calibrated Concept2 Model D ergometer. The test provides a valid criterion performance measure, with strong negative correlations with on-water rowing performance under standardized conditions (15, 56, 57, 69–73).

#### 2.3.2 Mental Toughness Index (MTI)

The MTI is an 8-item instrument that was designed to assess MT, commonly conceptualized as a psychological resource relevant to sustaining goal-directed functioning and performance under pressure, stress, or adversity. Responses use a 7-point Likert scale (*1* *=* *False, 100% of the time; 7* *=* *True, 100% of the tim*e), producing a composite score from 8 to 56. The MTI has demonstrated good internal consistency (Cronbach's *α* = 0.80–0.90) and validity across diverse athletic samples (58–65). A trained researcher delivered standardized instructions emphasizing honest, independent responses. Responses were collected electronically via secure tablets; coaches had no access to athletes’ reports or preferences. Because item-level data were not retained after composite scoring under field conditions, sample-specific internal-consistency reliability could not be computed; published *α* values in comparable athletic samples provide indirect support only. Temporal reliability of MTI composite scores was therefore evaluated using ICC (2,1) (two-way random, absolute agreement) across the four waves. The MTI was administered three times at each wave:

1. Athlete Self-Appraisal: Athletes rated their own MT using the standard MTI self-report format.
2. Perceived Head Coach Appraisal: Athletes completed the MTI again, this time responding to the items as they believed their head coach would rate them.
3. Perceived Assistant Coach Appraisal: Athletes completed the MTI a final time, responding as they believed their assistant coach would rate them.

The administration order was fixed across waves to preserve field consistency, although this design cannot rule out order or priming effects among the three MTI administrations.

#### 2.3.3 Perceived coach appraisals and preference

Following the three MTI administrations, athletes made a discrete choice: which coach they would prefer to consult for performance feedback at that wave. This single dichotomous-choice item was the primary self-reported outcome. Athletics department constraints prevented researchers from directly observing whether athletes actually consulted their selected coach, so this behavioral link could not be verified. *Δ*Alignment — the difference of absolute self–coach discrepancies used as the perceived alignment index — is a coarse proximity proxy with documented construct-validity limitations (47). The three RSA component scores (Self-MTI, Perceived Head MTI, Perceived Assistant MTI) and signed discrepancies are archived at Open Science Framework (OSF) (https://doi.org/10.17605/OSF.IO/FHJR9) for future researchers applying polynomial regression or Response Surface Analysis once adequate sample sizes are achieved.

### 2.4 Core unmeasured confounds

This pilot did not collect data on variables that may meaningfully shape coach preference in hierarchical NCAA team settings: coach role clarity, athlete–coach relationship quality (e.g., closeness, commitment), frequency of contact with each coach, perceived expertise of each coach, athlete team status (starter vs. reserve), interaction history, or designated functional coaching role (34). These are not secondary caveats; they are core design constraints that prevent isolation of SV processes from organizational role effects, interpreted further in §5.3. Future studies should explicitly measure these variables to better separate perceived alignment from organizational and relational explanations of coach preference.

## 3 Statistical analysis

Given the within-athlete outcome invariance discovered during data audit (§3.1), the primary analytical framework is cross-sectional and athlete-level. Two longitudinal sensitivity frameworks (GEE, GLMM) were fitted to the full 48-observation dataset exclusively as structural diagnostics to evaluate estimator behavior under outcome invariance — not for primary inference (74–76). These models were not used to increase inferential power or provide a primary hypothesis test. They were retained only to document how clustered estimators behaved under complete within-athlete outcome invariance and to inform future multi-team designs (18, 74). All statistical procedures were executed in R (Version 4.5.0) within the RStudio integrated development environment (Version 2025.05.0 + 496). To guarantee exact frequentist reproducibility, the random-number generator was initialized using *RNGkind(”L'Ecuyer-CMRG”)* with a fixed seed of *set.seed(2026)*. The complete de-identified data matrix, reproducible R scripts, and session logs are permanently archived on the OSF.

### 3.1 Primary athlete-level analysis

An initial data audit revealed that each athlete's self-reported coach preference was perfectly invariant across all four tracking waves, meaning individual preferences remained 100% constant over the competitive season. The planned longitudinal within-person analysis was therefore not informative because there was no within-athlete outcome variation to model. The effective sample size for evaluating the primary relational hypothesis was therefore established at the aggregated athlete level (*N* = 12 independent clusters) rather than across the 48 nominal observations. Primary estimation was conducted on these cross-sectional cluster means using three complementary analytical steps to maximize empirical precision:

1. Primary Athlete-Level Model: An athlete-level logistic regression model (generalized linear model with a binomial error family and a logit link function; *glm*) regressed the season-stable coach preference directly on season-mean *Δ*Alignment.
2. Estimation Companion: A Welch independent-samples *t*-test evaluated continuous season-mean *Δ*Alignment scores across the preference groups to accommodate unequal group sizes (*n* = 4 vs. *n* = 8) and variances. Small-sample corrected Hedges’ *g* served as the standardized effect size companion, tracking the absolute width of the 95% confidence interval (CI) to convey design insensitivity.
3. Construct Validity Audit: A Fisher's exact test evaluated the contingency matrix crossing the directional sign of season-mean *Δ*Alignment against the final choice to examine whether the relative proxy score tracked self-reported coach preference directionally.
4. Small-Sample Robustness Check: A Firth-penalized (bias-reduced) (77) logistic regression (*brglm2* package) (78, 79) was fitted as a robustness check on the primary logistic model, given the small, sparse outcome (4 head-coach-preference events among *N* = 12).

### 3.2 Longitudinal sensitivity models

Although primary estimation was governed by the aggregated athlete-level models, two longitudinal frameworks were fitted to the full 48-observation dataset to assess the behavior of clustered estimators under conditions of outcome invariance. These models were not used for primary inference or to increase statistical power. Instead, they were retained as structural diagnostics to document how GEE and GLMM estimators behaved when coach preference was invariant within athletes, and to inform future multi-team replications using data structures with sufficient within-athlete variation (18, 74).

A population-averaged GEE model was fitted using the *geepack* package (80). Because coach preference is within-athlete invariant, the exchangeable working correlation parameter approached its boundary limit (*α* ≈ 0.95), making the robust sandwich standard errors highly sensitive to software-specific solver choices. Consequently, GEE *p*-values were treated as estimator-dependent and unsuitable for hypothesis testing; inference rests strictly on evaluating the null stability of the point estimates (OR approximately 1.00 across all predictors) as a diagnostic confirmation of the athlete-level null profile.

A participant-specific random-intercept GLMM (binomial error family, logit link function) was executed via *lme4::glmer* using the *nlminb* optimizer method within the *optimx* package to guarantee mathematical convergence. Under conditions of perfect within-cluster outcome invariance, the GLMM experiences severe quasi-complete cluster-level separation (74–76), inflating the random intercept variance dramatically on the logit scale and rendering mixed-model standard errors uncalibrated and invalid for direct hypothesis testing. The specific structural GLMM formula was defined as: Coach Preference∼z ΔAlignment + *z* 2KTime + Time + (1 | Athlete ID). For all inferential checks, statistical significance was set at *α* = .05 (74, 75).

### 3.3 Perceived-alignment index construction and scaling

Data were structured in long format (*dplyr*). The primary perceived-alignment index, *Δ*Alignment was computed as: |MTI Self−MTI Perceived Head|−|MTI Self−MTI Perceived Assistant|. Negative values indicate closer perceptual proximity to the head coach; positive values indicate closer proximity to the assistant coach. Utilizing absolute discrepancies is theoretically grounded in SV theory's emphasis on cognitive congruence (23), but this difference-of-absolute-differences index has three documented limitations: (a) it collapses directional valence, making it impossible to distinguish SV (preference for accurate self-matching appraisals) from self-enhancement (preference for flattering overestimations); (b) it may exhibit reliability attenuation relative to raw component scores; and (c) it cannot model the non-linear congruence effects captured by polynomial regression and response surface analysis (47). The *Δ*Alignment index should therefore be interpreted as a coarse field proxy rather than a definitive operationalization of SV congruence. Raw component scores (self-, perceived-head-, and perceived-assistant-MTI) are archived at OSF to enable future RSA analyses.

Objective athletic performance was tracked using a maximal 2 K ergometer time trial recorded in total seconds, where a lower numeric value represents a faster, superior performance. This performance metric was included as a time-varying covariate to account descriptively for objective athletic performance as a potential alternative explanation for coach preference, addressing the possibility that coaching preferences may be influenced by physical performance rather than perceived self–coach alignment. For the longitudinal GLMM and GEE sensitivity models, both continuous predictors (ΔAlignment and 2 K Time) were *z*-standardized (mean = 0, SD = 1) to stabilize optimizer estimation and facilitate parameter comparison (81). The primary aggregated athlete-level models utilized raw, unstandardized season-mean parameters.

### 3.4 Model diagnostics

Prior to final parameter interpretation, the longitudinal models were subjected to a diagnostic pipeline to evaluate common estimation problems (82). Multicollinearity among fixed effects was evaluated using the Variance Inflation Factor (VIF). High-leverage data configurations were evaluated at the cluster level via an in-depth assessment of influential observations using Cook's distance, standardized DFBETAs, and a formal cluster-deletion significance test (*sigtest*) targeting the Athlete ID nesting node (83). Fixed-effects separation boundaries were evaluated using modern separation detection algorithms fitted via the *brglm2* package on a simplified, fixed-effects-only logistic structure.

### 3.5 *Post-hoc* design-sensitivity analysis

Because the available sample was constrained by the official varsity roster, a conventional prospective power analysis could not guide recruitment for this single-team pilot. We therefore used simulation-based design-sensitivity analyses to describe the current design and inform future multi-team planning. In accordance with guidelines for small-sample field pilots, we executed a tiered, post-hoc, simulation-based design-sensitivity analysis using the *simr* package in R (84, 85), comprising two tiers.

Tier A estimated power at the present *N* = 12, seeded from the separation-affected GLMM (random-intercept variance = 3,556), across a gradient of OR targets (1.5, 2.0, 3.0; 1,000 Monte Carlo iterations each, *α* = .05) informed by small-N sport-science recommendations (11, 67, 85). Because the generating model itself is degenerate, Tier A results are structural design-constraint illustrations rather than calibrated power estimates: power was flat across effect sizes (13.9%, 13.8%, and 13.5% for OR = 1.5, 2.0, and 3.0, respectively), consistent with a non-informative generating model. The absolute width of the small-sample corrected Hedges’ *g* confidence interval from the primary aggregated analysis (§3.1) remains the more reliable index of this study's own design sensitivity.

Tier B estimated power for prospective, pooled multi-team designs (*N* = 30, 50, 80 athletes) using a corrected generating model in which the random intercept was reset to the observed 33.3% head-coach-preference base rate (intercept = −0.693) and the random-intercept variance was set to correspond to an estimated ICC ≈ 0.15, informed by comparable coach-athlete relational cluster designs (86–91). This correction resolved an initialization artifact in which the separation-inflated intercept had previously been carried into extrapolation. Estimated power reached 66.0% (*N* = 30), 85.4% (*N* = 50), and 96.8% (*N* = 80) for OR = 2.0, and 92.6%, 99.4%, and 100% for OR = 3.0, respectively (Supplementary Table S2). These Tier B figures are planning-grade estimates suitable for prospective sample-size decisions in a multi-team replication; a formal prospective power analysis with a fully specified generating model is nonetheless recommended before any replication is launched.

### 3.6 Temporal stability analysis

As noted in §2.3.2, item-level response entries were not retained after composite scoring under field monitoring conditions, precluding sample-specific internal-consistency reliability (e.g., Cronbach's *α*). Temporal measurement reliability of the MTI composite scores across the season was therefore evaluated using the intraclass correlation coefficient within the *psych* package (92). Calculations utilized a two-way random-effects model evaluating single-rater absolute agreement [ICC (2,1)] as the primary stable index, alongside an average-measures companion [ICC (2,4)] to map general consistency across the entire four-wave competitive cycle. A two-way random-effects model was selected because all athletes were assessed at the same four fixed occasions (93, 94); one-way models, which do not partition shared wave-level variance, will generally yield modestly lower ICC estimates for the same data. For instance, a companion analysis of this cohort's mental toughness reliability using a one-way model reported a comparable estimate [ICC(1,1) = 0.66; (66)].

### 3.7 Harman single factor CMB screen

To assess common method bias arising from all perceptual measures being provided by the same rater (athlete) at the same time point (95, 96). Harman's single-factor test was conducted on the MTI composite scores across all three administration formats (Self-MTI, Perceived Head-MTI, Perceived Assistant-MTI) and four waves (*N* = 48 composite triplets). An unrotated principal components analysis examined whether a single unrotated factor explained the majority (> 50%) of total variance, which would indicate predominant common method variance. Because item-level data were not retained, this analysis operates on composite-level variance and should be interpreted as a conservative screen rather than a definitive bias assessment.

## 4 Results

The final sample consisted of 48 observations nested within 12 athletes. Table 1 provides the demographic summary, and Table 2 summarizes the analytical variables across the four measurement waves.

**Table 1:** Participant demographics (*N* = 12).

Variable	Age	Experience
Mean ± SD	20.75 ± 2.14	2.83 ± 3.07
Min	18	1
Max	24	10

**Table 2:** Descriptive statistics for analytical variables by wave.

Wave	N	*Δ*Alignment (mean ± SD)	2 K Time Performance (sec)	Head Coach Preference (*N*)	Head Coach Preference (%)
1	12	−2.08 ± 4.19	499.60 ± 27.50	4	33.33
2	12	−2.42 ± 3.99	496.27 ± 30.62	4	33.33
3	12	−1.83 ± 3.74	490.50 ± 26.61	4	33.33
4	12	0.00 ± 3.81	490.68 ± 27.44	4	33.33
Overall	48	−1.58 ± 3.92	494.26 ± 27.45	16	33.33

For *Δ*Alignment, negative values denote closer alignment with the head coach; positive values denote closer alignment with the assistant; values near 0 denote equal alignment. The sign does not indicate whether coach ratings exceed the self-rating.

### 4.1 Primary athlete-level analysis

**Figure 1:**
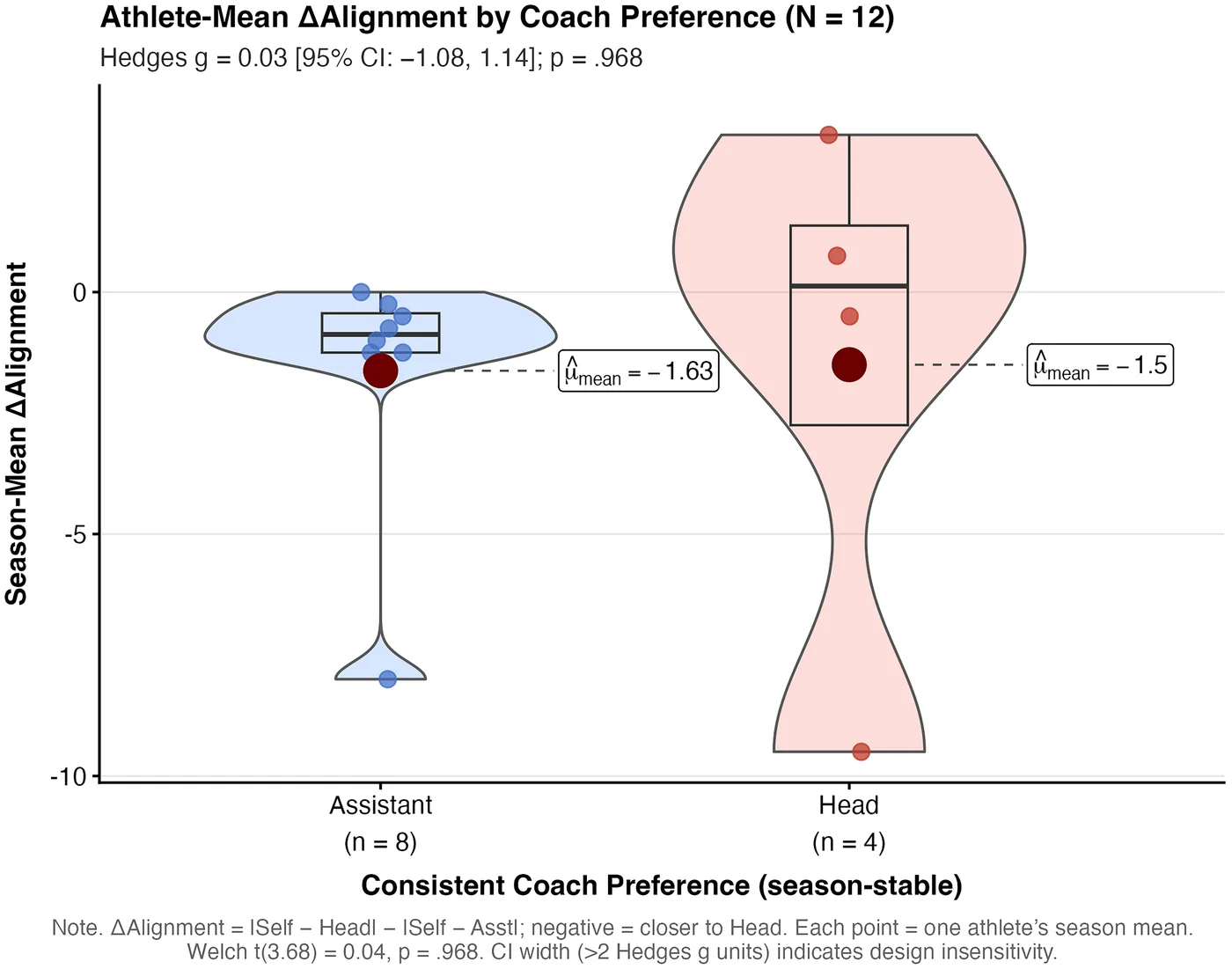
Athlete-Mean *Δ*Alignment by coach preference (*N* = 12). The violin/box plots display the distribution of each athlete's season-mean *Δ*Alignment score (averaged across the four competitive waves), grouped by season-stable coach preference (Assistant, *n* = 8: 7 closer to head coach, 1 closer to assistant coach; Head, *n* = 4: 2 closer to head coach, 2 closer to assistant coach). *Δ*Alignment = |Self – Perceived Head| – |Self – Perceived Assistant|; negative values indicate closer perceptual proximity to the head coach. A Welch independent-samples t-test returned a null, inconclusive result [Welch *t*(3.68) = 0.04, *p* = .968]. The standardized effect size was negligible [Hedges’ *g* = 0.03, 95% CI (−1.08, 1.14)]. The extremely wide confidence interval, spanning more than two Hedges’ *g* units, visually illustrates the design insensitivity inherent in a fixed, roster-restricted sample.

**Figure 2:**
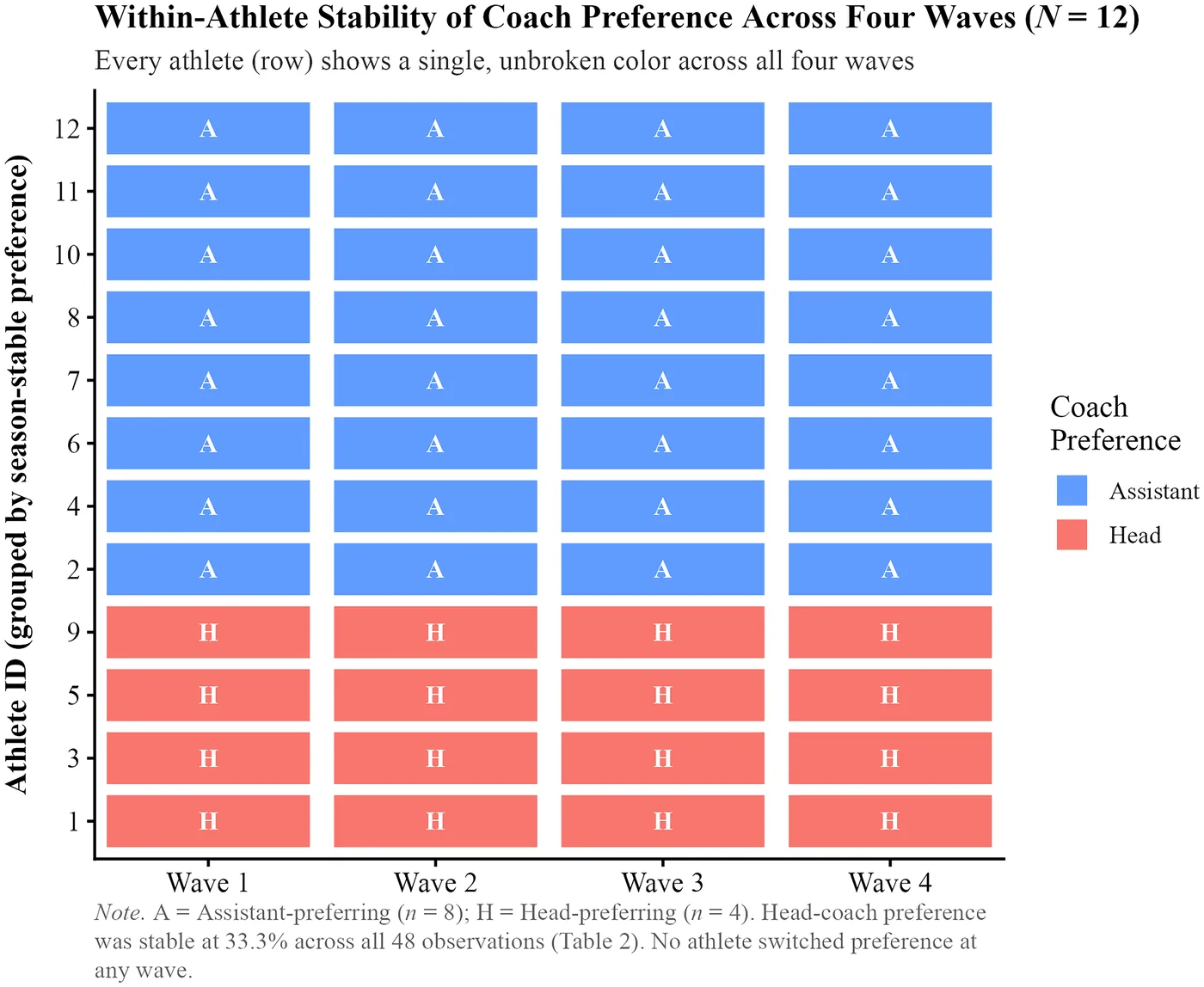
Within-Athlete stability of coach preference across four waves (*N* = 12). A = Assistant-preferring (*n* = 8); H = Head-preferring (*n* = 4). Head-coach preference was stable at 33.3% across all 48 observations (Table 2). No athlete switched preference at any wave.

As established in Statistical Analysis (§3.1), the complete invariance of self-reported coach preference within every athlete across all four waves reduced primary estimation to the aggregated athlete level (*N* = 12). An athlete-level logistic regression of season-stable preference on season-mean *Δ*Alignment returned OR = 1.01 [95% CI (0.71, 1.56), *p* = .95]. A Firth-penalized logistic regression, fitted as a small-sample robustness check, closely reproduced this estimate [OR = 0.99, 95% CI (0.70, 1.40), *p* = .969]. Supplementing the logistic model, a Welch independent-samples comparison of season-mean *Δ*Alignment by preference group (assistant: *M* = −1.63, *SD* = 2.62, *n* = 8; head: *M* = −1.50, *SD* = 5.56, *n* = 4) returned a mean difference of 0.13 [Head minus Assistant; 95% CI [−8.29, 8.54], Hedges’ *g* = 0.03, 95% CI [−1.08, 1.14]; Welch *t*(3.68) = 0.04, *p* = .968]. A Fisher exact test on the directionality of alignment by preference (where negative *Δ*Alignment = closer to head coach) was non-significant (*p* = .236). Notably, seven of the eight assistant-preferring athletes were perceptually closer to the head coach (negative *Δ*Alignment), while head-preferring athletes were split evenly (2 of 4 closer to the head coach). All athlete-level results showed no detectable association between season-mean *Δ*Alignment and self-reported coach preference (Table 3, Figure 1, Figure 2).

**Table 3:** Athlete-Level summary by coach preference (*N* = 12).

Characteristic	Assistant (*n* = 8) *N* = 8^1^	Head (*n* = 4) *N* = 4^1^	Difference (Assistant – Head)^2^	95% CI^2^	*p*-value^2^
Mean *Δ*Alignment (season average)	−1.63 (2.62)	−1.50 (5.56)	−0.13	−8.5, 8.3	0.968
Mean 2 K Time, s (season average)	499.90 (28.28)	482.99 (25.38)	17	−22, 55	0.331

^1^Mean (SD); ^2^Welch Two Sample t-test; CI, Confidence Interval

Descriptive athlete profiles reveal the following patterns across the two preference groups. Among assistant-preferring athletes (*n* = 8), season-mean self-MT ranged from 33 to 48 (*M* ≈ 41); season-mean perceived head-coach MT and perceived assistant-coach MT were broadly similar, with seven of eight athletes displaying negative *Δ*Alignment, indicating closer perceived proximity to the head coach despite preferring the assistant. Among head-preferring athletes (*n* = 4), season-mean self-MT ranged from 36 to 50 (*M* ≈ 43); two athletes displayed negative *Δ*Alignment and two displayed positive *Δ*Alignment, consistent with the uniformly null directional pattern. Mean 2 K times were descriptively faster for head-preferring athletes (*M* = 482.99 s) than assistant-preferring athletes (*M* = 499.90 s), though this difference was small and non-significant (Welch *p* = .331).

### 4.2 Longitudinal sensitivity models (diagnostic)

Full model outputs are provided in Supplementary Table S1. Model diagnostics did not indicate problematic multicollinearity (VIFs all < 1.4; z Delta = 1.33, z Time2 K = 1.07, Time = 1.31) or influential observations (Cook's D < 0.001 for all 48 observations). The population-average GEE returned uniformly null point estimates (*Δ*Alignment OR = 1.00; 2 K Time OR = 1.00; all wave contrasts OR = 1.00; QIC = 68.62). The GLMM exhibited quasi-complete cluster-level separation (random intercept variance = 3,556; SD = 59.6), rendering its standard errors uncalibrated; GLMM Marginal *R*^2^ = .000, Conditional *R*^2^ = .999 (both expected under null fixed effects and separation-inflated random effects). Given the exchangeable boundary correlation (GEE *α* = 1.0) and GLMM separation, *p*-values from these models are not interpreted. These outputs are reported only as diagnostics of estimator behavior under outcome invariance; primary estimation remains the athlete-level analysis.

#### 4.2.1 Tiered design-sensitivity analysis

Tier A simulation-based design sensitivity (1,000 iterations per scenario) (85) produced illustrative power values of 13.9% [95% CI (11.8%, 16.2%)] for OR = 1.5, 13.8% [95% CI (11.7%, 16.1%)] for OR = 2.0, and 13.5% [95% CI (11.4%, 15.8%)] for OR = 3.0. Tier B extrapolation, using the corrected generating model described in §3.5, estimated power for pooled multi-team designs at 66.0% (*N* = 30), 85.4% (*N* = 50), and 96.8% (*N* = 80) for OR = 2.0, and 92.6% (*N* = 30), 99.4% (*N* = 50), and 100% (*N* = 80) for OR = 3.0. Tier A and Tier B values are tabulated in full in Supplementary Table S2 and the power curve is graphically presented in Supplementary Figure 1.

### 4.3 Temporal stability analysis

Temporal stability across the four waves was moderate to good overall: MTI self-appraisal ICC(2,1) = 0.685 [95% CI (0.424, 0.879)] – ICC(2,4) = 0.897 [95% CI (0.746, 0.967)]; perceived assistant-coach appraisal ICC(2,1) = 0.883 [95% CI (0.567, 0.967)] – ICC(2,4) = 0.968 [95% CI (0.840, 0.992)]; and the *Δ*Alignment difference score ICC(2,1) = 0.726 [95% CI (0.482, 0.897)] – ICC(2,4) = 0.914 [95% CI (0.788, 0.972)]. Perceived head-coach appraisal was comparatively less stable, ICC(2,1) = 0.565 [95% CI (0.167, 0.838)] – ICC(2,4) = 0.839 [95% CI (0.446, 0.954)]; because the ICC(2,1) lower bound (.167) falls in the poor-reliability range (<.40) (92), temporal stability of perceived head-coach appraisals is more accurately characterized as moderate with wide confidence intervals rather than uniformly good.

### 4.4 Harman common method bias screen

Harman's single-factor test, conducted via unrotated principal components analysis on the three MTI composite scores (self-, perceived-head-, and perceived-assistant-appraisal) across all 48 athlete-wave observations, yielded a first unrotated factor explaining 61.3% of total variance among the three composites, exceeding the 50% threshold conventionally used to flag predominant common method variance (95, 96). Because item-level responses were not retained, this composite-level screen cannot provide a definitive common-method-bias adjustment, and item-level bias-adjustment analyses were not possible (see Limitations).

## 5 Discussion

### 5.1 Primary interpretations and design sensitivity constraints

This pilot deployed a brief self-coach alignment audit in a live NCAA rowing environment. The within-athlete outcome invariance — all 12 athletes maintained identical preferences across all four waves — prevented meaningful longitudinal inference and reduced primary estimation to a cross-sectional athlete-level comparison (*N* = 12). The primary athlete-level analysis and both diagnostic longitudinal sensitivity models converged on a uniformly null profile: perceived MT alignment showed no detectable association with self-reported coach preference in the primary athlete-level analysis — a null profile corroborated by a Firth-penalized robustness check on the primary model — with diagnostic longitudinal models showing the same null point-estimate pattern (11, 36, 37, 74, 77, 78, 80, 97). We emphasize that this null finding does not constitute evidence against SV theory; it constitutes evidence that the current design lacked the sensitivity to evaluate SV theory, a critically important distinction (22, 98).

The flat Tier A power curve (13.5–13.9% across OR = 1.5–3.0) confirms the degenerate generating model provides no effect-size information, making this single-team study design structurally incapable of providing meaningful evidence for or against the SV hypothesis; because the model was seeded from a separation-affected GLMM (random-intercept variance = 3,556, SD = 59.6) — a mathematical artifact of perfect within-subject outcome invariance rather than an index of substantive between-athlete heterogeneity — no minimum-detectable-effect grid search could be meaningfully derived from it either (22, 74–76, 99). Tier B planning estimates offer the more actionable takeaway: pooling approximately 50 athletes across four to five rosters would achieve 85% power for a moderate alignment–preference association (OR = 2.0), providing a concrete design target for future multi-team collaboration. Publishing transparent, open-science null results from specialized, roster-restricted collegiate cohorts remains essential for establishing accurate effect-size priors and preventing future researchers from deploying underpowered longitudinal designs (11, 13, 14, 18, 36, 37, 43, 97–102).

### 5.2 Methodological gaps and construct validity critiques

A construct validity insight independent of statistical power comes from the directional contingency matrix (24, 47). Seven of the eight assistant-preferring rowers were perceptually closer to the head coach (negative season-mean *Δ*Alignment), while head-preferring rowers were split evenly (2 of 4 closer to the head coach). Fisher's exact test provided no evidence that this discrepancy index tracked preference directionally (*p* = .236), consistent with the limitation already noted in §3.3 — as a difference of absolute discrepancies, *Δ*Alignment measures relative proximity but cannot separate SV (preference for accurate confirmation) from self-enhancement (preference for favorable evaluation, regardless of accuracy). In highly driven, competitive cohorts, selecting a coach perceived to overestimate one's capabilities could reflect self-enhancement striving rather than accurate SV (24, 103).

Response Surface Analysis (RSA) (104–106) is the methodologically superior approach for congruence hypotheses, modeling the non-linear congruence surface through second-order polynomial terms that cleanly separate similarity effects (the core SV prediction) from favorable-rating bias (self-enhancement). This analytical approach was not implemented here: applied to a binary preference outcome, it requires 5–6 free parameters, and *N* = 12 athletes with only 4 head-preference events yields approximately 0.8 events per parameter — far below the ≥80–100 observations-per-parameter benchmark (104, 106). Applying RSA to the present data would produce numerically incoherent, not merely imprecise, surfaces. Future multi-team collaborations pooling ≥80 athletes with roughly balanced preference outcomes can apply RSA directly to the raw component scores (MTI_Self, MTI_Perceived_Head, MTI_Perceived_Assistant) already archived at OSF (106–108).

Common method bias is a related structural concern, since all perceptual measures were self-reported by athletes alone. The composite-level Harman's screen (§4.4) found the first unrotated factor explained 61.3% of variance among the three MTI composites, exceeding the conventional 50% threshold; because *Δ*Alignment is itself a difference score between two of these correlated composites, elevated shared-method variance would be expected to attenuate rather than inflate its association with preference, so this screen cannot fully rule out CMB as a contributor to the null finding, and item-level bias-adjustment analyses were not possible because item-level responses were not retained (95, 96). Future designs should retain item-level data and obtain direct coach MTI ratings, enabling a two-layer SV test: whether perceived congruence (athlete meta-perception) predicts preference, and whether actual congruence (athlete–coach dyadic match) predicts preference independently of or in interaction with perceived congruence (32, 109–113).

### 5.3 Fixed organizational realities versus fluid psychological choices

The complete season-long time invariance of coach preference points to a parsimonious structural alternative (107, 108). Self-verification theory was developed and validated within civilian, voluntary settings involving chosen interpersonal dyads (24, 33, 114, 115), whereas competitive intercollegiate sport operates within assigned, highly regulated hierarchies with fixed authority structures and rigid organizational roles (108, 116–118). None of these organizational variables were measured here (§2.4), but the complete temporal stability of preference across four waves — even as *Δ*Alignment itself varied (wave means ranging from −2.42 to 0.00) — is consistent with athletes nominating a preferred coach based on baseline familiarity, program authority, or designated technical/developmental role, rather than perceived MT alignment (32, 107, 108, 119). Coach preference may also reflect accumulated relational history, including prior interpersonal experiences, emotional bonds, and trust developed over time, independently of both perceived MT alignment and formal organizational hierarchy. Performance offered a related structural cue: head-coach-preferring rowers were descriptively faster (M 2 K = 482.99 s vs. 499.90 s for assistant-preferring athletes), though the difference was small and non-significant (Welch *p* = .331), consistent with the uniformly null profile across all estimators. Independent modeling of this cohort's 2 K trajectories confirms genuine within-season performance improvement (66), supporting 2 K time's inclusion here as a meaningful, non-static covariate.

Without measures of coach role clarity, frequency and type of athlete–coach contact, interpersonal trust, perceived expertise, designated technical vs. motivational coaching function, and athlete status (starter vs. reserve), the present null result remains ambiguous between a genuine absence of SV effects and a structural masking of those effects by organizational hierarchy (33–35, 107, 108, 120–123). Future designs should measure and model these variables to better distinguish perceived alignment from institutional and relational explanations of coach preference.

### 5.4 Theoretical contributions, applied pillars and translation

Despite this design insensitivity, this pilot highlights important methodological constraints for future research applying SV dynamics in sport psychology (124, 125). Historically, SV research has been confined to dyadic tracking within educational, clinical, and traditional corporate workplaces (24, 33, 114, 115, 126). This pilot extends the foundation into an NCAA rowing context defined by high physical strain, structural interdependence, and complex rater dynamics (15, 42, 127). Using MT as the evaluative domain addresses long-standing criticisms of the MT literature's reliance on cross-sectional, male-dominated samples, while ignoring socialized relational networks (34, 41, 44, 108). Moreover, a female collegiate rowing roster helps diversify the field's empirical base (15, 42, 43, 101, 127), while positioning MT not as an isolated trait, but as a psychological resource embedded within social environments, consistent with Conservation of Resources theory's concept of resource caravan passageways (26, 128). The present null results do not confirm that athletes seek environments validating their perceived resources, but this expectation is grounded in motivational climate research showing relational contexts shape perceived competence and wellbeing in elite sport (107, 129–131).

From a translational perspective, this pilot offers a roadmap for converting abstract psychosocial theory into an in-season, low-burden tracking workflow (2, 5, 45, 46, 132). The complete stability of preference across four waves raises the possibility that a single preseason administration may suffice to characterize an athlete‘s relational pattern, meaningfully reducing measurement burden during the competitive calendar — but the *Δ*Alignment score‘s predictive validity remains undemonstrated in the current dataset, and this inference requires replication with larger, more diverse samples before any practical recommendation can be made (133, 134). If validated in larger and better-measured samples, this audit could eventually help coaching staffs identify how athletes perceive coach feedback relationships (5, 135). This could contribute to what Keegan et al. termed ‘theories of practice’, frameworks that are scientifically grounded and immediately usable in the training environment (136, 137), and answers recent calls to co-produce sport psychology knowledge with ‘a foot in both camps’ of research and coaching (6, 133). As Sarkar argued, the field's future lies in translating constructs into tools concise enough for tomorrow's training session yet rigorous enough to withstand scholarly scrutiny (2), enriching positive psychology's empirical base in applied sporting contexts (46). This work shows that the alignment audit was feasible under the conditions of this single-team pilot and could be deployed with minimal burden during a competitive season; the GEE and GLMM frameworks were not interpretable in this dataset due to outcome invariance, and their contribution here is as a structural diagnostic, not as a validated model of SV processes.

### 5.5 Methodological limitations and future replications

The primary strength of this study is its ecological validity: data were collected during actual NCAA championship competition, capturing genuine coach-athlete decisions under competitive pressure (102). Convergence across the primary athlete-level and both sensitivity estimators confirms the null profile is not a modeling artifact, and the diagnostic pipeline (VIF, Cook's D, and separation testing), verified model integrity (82–84, 138, 139).

Four limitations bound interpretation. First, all perceptual data were athlete-reported; actual coach MTI ratings were not collected, preventing any test of objective congruence. This is theoretically defensible for SV specifically, since the theory concerns subjective social reality rather than objective accuracy but a genuine two-layer test — perceived versus objective congruence — requires parallel coach ratings this design did not collect. Second, the organizational and relational confounds most likely to determine coach preference in an NCAA setting (§5.3) were not measured, leaving the null association ambiguous between a genuine absence of SV effects and structural masking by institutional hierarchy. Third, common method bias is a structural concern given athlete-only self-report, and item-level MTI data were not retained, precluding *post-hoc* CMB detection beyond the composite-level Harman's screen (§5.2). Fourth, *Δ*Alignment is a relative-proximity difference score that cannot distinguish SV from self-enhancement — an operationalization gap that should be resolved via RSA before any powered replication (11, 14, 32, 33, 36, 38, 47, 68, 95, 96, 106, 140–147).

The highest-priority next step is a mixed-methods multi-team replication synthesizing the design changes identified above: pool data across approximately 50–80 athletes from four to five NCAA teams spanning Division I and Division II — Supplementary Table S2 indicates 85%–97% power for a moderate effect (OR = 2.0) across this range (§5.1) — replace *Δ*Alignment with RSA using the OSF-archived component scores (§5.2), measure the organizational confounds identified in §5.3 as covariates, and retain item-level MTI data while obtaining direct coach ratings to support the two-layer congruence and CMB tests described in §5.2. A qualitative component adding brief semi-structured post-season athlete interviews — probing which coach provides MT-relevant feedback, interaction frequency on psychological topics, and whether trust or authority hierarchy drove their preference — would enable triangulation between metric indices and preference narratives, directly addressing the organizational-confounding alternative the quantitative data alone cannot resolve (106, 111, 148–154). Future work should also model additional moderators of the SV–preference link, such as athlete personality (e.g., need for cognition) and team climate (cohesive vs. conflicted) (155–157). This pilot serves primarily as an empirical benchmark for the sample sizes and effect magnitudes future replications should plan around (13, 18, 84, 99, 138, 158, 159).

## 6 Conclusion

This field-based exploratory study deployed a brief self-coach alignment audit in a live NCAA rowing environment and found no evidence that perceived MT alignment was detectably associated with self-reported coach preference. This null finding is inconclusive rather than theory-disconfirming: given a Tier A design that provided no effect-size sensitivity at *N* = 12, it reflects the limits of a single-team pilot rather than evidence against self-verification theory. The primary contribution is methodological — a reproducible, low-burden audit template, an open-science data and code archive, and a tiered design-sensitivity framework (Supplementary Table S2) that provides preliminary sample-size planning estimates for future multi-team studies rather than a *post-hoc* power estimate alone. This work demonstrates that the alignment audit itself can be deployed quickly and with minimal burden in a competitive-season setting; the GEE and GLMM analyses served only as structural diagnostics of estimator behavior under outcome invariance, not as validated tests of SV processes. In doing so, this study contributes to narrowing the research-practice gap by clarifying the logistical and statistical hurdles, most notably the necessity of *a priori* power planning that a powered multi-team replication must address. It serves as a call for exactly that: larger-scale, collaborative research that can more adequately test whether SV processes operate in competitive sport coach-athlete relationships.

## Data Availability

The datasets presented in this study can be found in online repositories. The names of the repository/repositories and accession number(s) can be found below: https://doi.org/10.17605/OSF.IO/FHJR9.
