# Supplementary material for: A pilot self-coach alignment audit in NCAA collegiate rowers: field feasibility, null findings, and design-sensitivity analysis for future self-verification research
Source: Front Sports Act Living. 2026 Aug 25;8:1902953. doi: 10.3389/fspor.2026.1902953 (PMC13552130; doi:10.3389/fspor.2026.1902953)
Supplement: Supplementary file 1 [file Table1.docx]

Supplementary Table S1. Parameter Estimates for the GLMM and GEE Predicting Coach Preference (Diagnostic Only — Not Used for Primary Inference). GLMM columns reflect the participant-specific random-intercept model; its random-intercept variance (3556, SD = 59.6) is separation-inflated by within-athlete outcome invariance, so GLMM ORs and p-values are diagnostic only. GEE columns reflect the population-averaged model at the exchangeable working-correlation boundary (alpha = 1.0); GEE p-values are estimator-dependent at this boundary and are not used for hypothesis testing — only the near-null GEE point estimates (OR) are interpreted, as reported in the main text. GLMM AIC = 26.8, BIC = 39.9. GEE QIC = 68.6.

| Predictor | *b* (GLMM) | SE (GLMM) | *z* (GLMM) | *p* (GLMM) | OR (GLMM) | GLMM CI low | GLMM CI high | OR (GEE) | GEE CI low | GEE CI high | *p* (GEE) |
| --- | --- | --- | --- | --- | --- | --- | --- | --- | --- | --- | --- |
| (Intercept) | -12.966 | 6.192 | -2.094 | 0.036 | 0.00 | 0 | 0.44 | 0.5 | 0.15 | 1.66 | 0.258 |
| z_Delta | -0.200 | 4.534 | -0.044 | 0.965 | 0.82 | 0 | 5,927.71 | 1.0 | 1.00 | 1.00 | 0.112 |
| z_Time2K | -0.800 | 3.641 | -0.220 | 0.826 | 0.45 | 0 | 564.92 | 1.0 | 1.00 | 1.00 | 0.056 |
| Time2 | -0.141 | 6.198 | -0.023 | 0.982 | 0.87 | 0 | 164,062.02 | 1.0 | 1.00 | 1.00 | 0.806 |
| Time3 | -0.207 | 6.105 | -0.034 | 0.973 | 0.81 | 0 | 127,904.60 | 1.0 | 1.00 | 1.00 | 0.173 |
| Time4 | -0.113 | 6.461 | -0.018 | 0.986 | 0.89 | 0 | 282,184.68 | 1.0 | 1.00 | 1.00 | 0.238 |
