# Supplementary material for: A pilot self-coach alignment audit in NCAA collegiate rowers: field feasibility, null findings, and design-sensitivity analysis for future self-verification research
Source: Front Sports Act Living. 2026 Aug 25;8:1902953. doi: 10.3389/fspor.2026.1902953 (PMC13552130; doi:10.3389/fspor.2026.1902953)
Supplement: Supplementary file 2 [file Table2.docx]

Supplementary Table S2. Tiered Simulation-Based Design-Sensitivity Analysis (Tier A: Structural Illustration at N = 12; Tier B: Multi-Team Planning Extrapolation). Tier A seeded from the separation-affected GLMM (random-intercept variance = 3,556); values are structural design-constraint illustrations, not calibrated power estimates. Tier B uses a corrected generating model (intercept reset to the observed 33.3% base rate; ICC ≈ 0.15) and provides planning-grade estimates for prospective multi-team sample-size decisions.

| Tier | *N* (athletes) | Target OR | Power (%) | 95% CI |
| --- | --- | --- | --- | --- |
| Tier A (structural) | 12 | 1.5 | 13.9 | [11.8, 16.2] |
| Tier A (structural) | 12 | 2.0 | 13.8 | [11.7, 16.1] |
| Tier A (structural) | 12 | 3.0 | 13.5 | [11.4, 15.8] |
| Tier B (planning) | 30 | 2.0 | 66.0 | [61.7, 70.1] |
| Tier B (planning) | 50 | 2.0 | 85.4 | [82, 88.4] |
| Tier B (planning) | 80 | 2.0 | 96.8 | [94.9, 98.2] |
| Tier B (planning) | 30 | 3.0 | 92.6 | [89.9, 94.7] |
| Tier B (planning) | 50 | 3.0 | 99.4 | [98.3, 99.9] |
| Tier B (planning) | 80 | 3.0 | 100.0 | [99.3, 100] |
